# Bacteremia and resistant gram-negative pathogens among under-fives in Tanzania

**DOI:** 10.1186/1824-7288-39-27

**Published:** 2013-05-08

**Authors:** Alexandra Christopher, Stephen E Mshana, Benson R Kidenya, Aldofineh Hokororo, Domenica Morona

**Affiliations:** 1 Department of Pediatric and Child Health, Weill School of Medicine, CUHAS-Bugando, Mwanza, Tanzania; 2 Department of Microbiology/Immunology Weill School of Medicine, CUHAS-Bugando, BOX 1464, Mwanza, Tanzania; 3 Department of Biochemistry and Molecular Biology, Weill School of Medicine, CUHAS-Bugando, Mwanza, Tanzania; 4 Department of Parasitology/Entomology Weill School of Medicine, CUHAS-Bugando, Mwanza, Tanzania

## Abstract

**Background:**

Antibiotic resistance is one of the most serious public health concerns worldwide and is increasing at an alarming rate, making daily treatment decisions more challenging. This study is aimed at identifying local bacterial isolates and their antimicrobial susceptibility patterns to avoid irrational antibiotic use, especially in settings where unguided management occurs and febrile illnesses are predominant.

**Material and methods:**

A hospital-based prospective cross-sectional study was conducted from September 2011 to February 2012. Febrile children were serially recruited and demographic and clinical data were collected using a standardized data collection tool. A blood culture was performed and identification of the isolates was undertaken using in-house biochemical tests. Susceptibility to common antibiotics was investigated using the disc diffusion methods.

**Results:**

Of the 1081 children admitted during the study period, 317 (29.3%) met the inclusion criteria and were recruited, of whom 195 (61.5%) and 122 (38.5%) were male and female respectively. The median age was 18 months with an interquartile range of 9 to 36 months. Of the 317 children, 251 (79.2%) were below or equal to 36 months of age. The prevalence of bacteremia was 6.6%. A higher prevalence of bacteraemia was observed in children below 36 months than in those ≥ 36 months (7.5% vs. 3.0%, p = 0.001). Predictors of bacteraemia were an axillary temperature of >38.5 °C (OR =7, 95% CI = 2.2 - 14.8, p-value = 0.0001), a positive malaria slide (OR =5, 95% CI = 3.0 - 21.2, p-value = 0.0001) and a high neutrophils’ count (OR =21 95% CI = 5.6 - 84, p-value = 0.0001). *Escherichia coli* and *Klebsiella pneumoniae* accounted for 7 (33.3%) and 6 (28.6%) of all the isolates respectively. Others gram-negatives bacteria were *Citrobacter* spp 2 (9.5%), *Enterobacter* spp 1 (4.25%)*, Pseudomonas* spp 2 (9.5%), *Proteus spp* 1 (4.25%) and *Salmonella spp* 1 (4.25%)*.* These isolates were highly resistant to ampicillin (95%), co-trimoxazole (90%), tetracycline (90%), gentamicin (80%), augmentin (80%), chloramphenicol (65%), ceftriaxone (35%), cefotaxime (35%) ciprofloxacin (30%), amikacin (30%), ceftazidime (25%) and norfloxacine (10%).

**Conclusion:**

Multi-resistant gram-negative bacteria are the commonest cause of bacteremia in under-fives attending the Bugando Medical Centre, Mwanza, Tanzania. A high body temperature, a positive malaria slide and a high absolute neutrophils’ count were all independent risk factors found to predict bacteremia. A higher mortality rate was observed in children with bacteraemia. Continuous epidemiological surveillance should be conducted so that a proper and effective antibiotics management can be instituted, especially in children with a high grade fever, a positive malaria slide and a high neutrophils’ count.

## Background

Febrile illnesses remain the leading cause of morbidity and mortality among children, especially in Sub-Saharan Africa [1,2]. The World Health Organization (WHO) defines febrile illness as an acute illness characterized by a rise in body temperature. Viral infections, bacteremia and malaria are among the commonest causes of these febrile illnesses in the developing countries [3]. Moreover, a high mortality rate is associated with bacteraemia. A study done at the Muhimbili National Hospital in Tanzania reported a mortality of 40% in pediatric patients with laboratory confirmed bacteremia [4]. Worldwide, 76% (4.6 million) of the underfives deaths occur due to undiagnosed invasive bacterial infections ( http://www.unicef.org/publications/files/2004_OfficialSumm_ENG.pdf). In malaria endemic areas, 11% of the children admitted with fever are found to have bacteremia. Twelve percent (12%) of those children will die because malaria was over diagnosed at the expense of other causes of fever [5]. Moreover, bacterial culture to diagnose bacterial infection is not routinely done in most of the primary and secondary health facilities. This leads to unguided empirical treatment with antibiotics, which, in turn, may result into an increase of antimicrobial resistance [6].

Varieties of bacteria have been found to cause febrile illnesses in children. These include *Staphylococcus spp*, *Streptococcus* spp*, Enterobacter* spp, *Escherichia coli*, *Klebsiella pneumoniae*, *Pseudomonas* spp*, Enterococcus* spp*, Neisseria meningitides, Salmonella* spp*, Moraxella catarrhalis, Haemophilus influenzae* and *Campylobacter spp*[7-9]. The WHO claims that inadequate access to appropriate near-patient diagnostic tools can also be a driving factor for prescribing antibiotics when not clearly indicated [10]. The findings from this study will contribute to a proper implementation of the guided empirical treatment of common bacteria isolates causing febrile illnesses in our setting. Predictors of positive blood culture and of in-hospital mortality are crucial to clinicians to ensure a timely and appropriate management response.

## Methods

This was a hospital-based prospective cross-sectional study including 317 children aged 2 to 60 months admitted at the Bugando Medical Centre (BMC) general pediatric ward. A total of 80% of the children were referred from other health facilities in the Lake Zone, North_Western Tanzania, during the study period (September 2011 to February 2012). Children enrolled in the study were those aged between 2–60 months with an axillary temperature ≥ 37.5 °C at the time of admission. Children with a history of antibiotics’ use in the past 24 hours were also included into the study. All children included in the study had informed parent/guardian consent.

### Sample size estimation and sampling

The sample size was calculated by the double proportional formula for predictors using STATA version 11. The two proportions used were based on studies undertaken at the General Pediatric wards, Kenyatta National Hospital, Nairobi, Kenya [11] which showed a 13.2% prevalence of bacteremia among children without malaria, and in Boston, Massachusetts, USA, which looked into fever as a predictor of bacteremia and found the prevalence to be 4% [12]. The minimum sample size required was 270 patients but the study enrolled 317 patients. All children aged between 2 to 60 months attending the Bugando Medical Centre were serially recruited until the sample size was reached.

### Sample collection and laboratory procedures

Two blood specimens from the same venepuncture were collected from all enrolled children. The child’s skin was cleaned with 70% ethanol and allowed to dry before blood was drawn for culture and other investigations. For the blood culture, the ratio of blood: Brain Heart Infusion broth (Oxoid UK) was 1:10. Two bottles of Brain Heart Infusion broth with the collected blood specimen were transported to the laboratory and incubated for 18–24 hours at 37°C aerobically. The specimens were sub-cultured on blood, MacConkey and chocolate agar, as previously described [13]. For positive blood cultures, the isolates were identified according to conventional physiological and biochemical methods [13,14]. The antimicrobial susceptibility patterns of all isolates were determined by the disc diffusion method according to the Clinical Laboratory Standards Institute [15]. Antimicrobial susceptibility patterns for gram negative bacteria were ampicillin, gentamicin, norfloxacin, amoxicillin/clavulanic acid, co-trimoxazole, chloramphenicol, ceftriaxone, ceftazidime and meropenem. All isolates resistant to third generation cephalosporin were tested for Extended Spectrum Beta-Lactamases (ESBL) production using the disk approximation method. To determine ESBL production, ceftazidime (30 μg) and cefotaxime (30 μg) discs were placed equidistant from the amoxycillin/clavulanate (20/10 μg) disc; an enhanced zone of inhibition towards amoxycillin/clavulanate (20/10 μg) disc was considered as a positive result for ESBL production [16,17]. The Bugando Medical Centre laboratory is participating in a bacteriology external quality assurance coordinated by a reference laboratory in South Africa (WHO/NICD). For the quality control of the media, discs and incubation conditions, we used *Escherichia coli* ATCC 25922, *Streptococcus pneumoniae* ATCC 49619 and *Staphylococcus aureus* ATCC 25923. Total white blood cells and neutrophils’ counts were determined using a haematology analyzer (Beckman Coulter (UK) Ltd). HIV and malaria were diagnosed as previously described [18,19].

### Data analysis

Data collected were entered into a computer using EpiData version 3.1 (CDC, Atlanta, USA) according to codes given, and analyzed using the STATA version 11 (College Station, Texas, USA). Categorical variables were summarized as proportions and were analyzed using the Pearson’s Chi-square test to observe the differences among the various groups. Continuous variables were summarized as mean or median and were analyzed using the student t-test or Wilcoxon-Mann–Whitney test where appropriate. Univariate analysis and multivariate logistic regression models were fitted to determine the predictors of culture positivity and in-hospital mortality. Predictors with p-value less than 0.2 were fitted into the multivariate logistic regression analysis and their odds ratios and 95% confidence interval were noted. Predictors with p-value of less than 0.05 were considered statically significant. Dependent variables included bacteremia and in-hospital mortality while independent variables were the clinical presentation, the laboratory findings and the socio-demographic characteristics of the patient. The study was approved by the BMC/CUHAS ethics committee and parents/caretakers were asked to sign an informed consent form.

## Results

**Figure 1:**
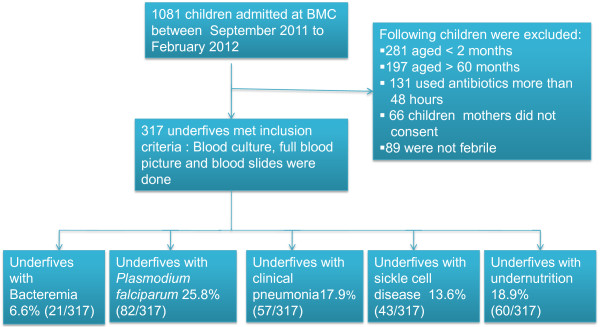
Flow chart showing children studied and magnitude of different outcome.

During the study period between September 2011 and February 2012, a total of 1081 infants and children were admitted to the BMC paediatric ward. A total of 317(40.6%) met the inclusion criteria and were studied (Figure 1). Of these, 195(61.5%) were male. The median age was 18 months with an interquartile range of 9 to 36 (Table 1). All children recruited in this study received vaccines as recommended by the Tanzania Immunization Programme. Vaccines provided by the programme include the BCG and OPV 0 at birth; the OPV-1 and DPTHB-Hib 1 at 4 week; the OPV-2 and DPTHB-Hib 2 at the 8th week, and the OPV-3, DPTHB-Hib 3 at the 12th week. The measles vaccine is provided at 9 months.

**Table 1:** Showing demographic characteristics

**Characteristics**	**n (%)**
**Age (months)**	
≤36	251 (79.2)
>36	66 (20.8)
**Sex**	
Male	195 (61.5)
Female	122 (38.5)
**Malnutrition**	
No	257 (81.1)
Yes	60 (18.9)
**Antibiotic use less than 24 hrs**	
No	70 (22.2)
Yes	247 (77.8)
**Sickle cell disease**	
No	274 (86.4)
Yes	43 (13.6)
**Congenital heart disease**	
No	308 (97.2)
Yes	9 (2.8)
**Hydrocephalus**	
No	311 (98.1)
Yes	6 (1.9)
**HIV Positive**	
No	301 (94.9)
Yes	16 (5.1)

The prevalence of bacteremia in the present study was 6.6%. Most of the children with bacteremia had an age less than or equal to 36 months (7.5% vs. 3.0%, p > 0.05). Ten blood cultures (3.2%) showed growth in only one bottle on the first culture and all were negative on repeat blood culture. These were regarded as contaminants and, since they may have masked a true infection, the respective patients were excluded from the analysis. All contaminants were coagulase negative Staphylococci.

### Bacteria isolates and antimicrobial susceptibility pattern

During the study, common bacteria isolated were *Escherichia coli* and *Klebsiella pneumoniae,* which *accounted* for 7 (33.3%) and 6 (28.6%) of total isolates respectively. Others gram-negatives isolated were *Citrobacter* spp 2 (9.5%), *Enterobacter* spp 1 (4.25%)*, Pseudomonas aeruginosa* 2 (9.5%), *Proteus* spp 1 (4.25%) and *Salmonella* spp 1 (4.25%)*.* Only one isolate was gram-positive bacteria and was identified as *Staphylococcus aureus.* Gram-negative enteric bacteria were resistant to most of the antibiotics tested, including ampicillin (95%), co-trimoxazole (90%), tetracycline (90%), gentamicin (80%), augmentin (80%), chloramphenicol (65%), ciprofloxacin (30%), amikacin (30%), ceftazidime (25%), ceftriaxone (35%), cefotaxime (35%) and norfloxacin (10%) (Table 2). Twenty seven percent of the gram-negative enteric bacteria were found to produce extended spectrum beta lactamase (ESBL) using the disk approximation method.

**Table 2:** susceptibility pattern of 20 gram negative isolates from febrile children (N = 317)

**NO**	**ISOLATE**	**G**	**CIP**	**TET**	**AK**	**AMC**	**NO**	**C**	**SXT**	**A**	**CZA**	**CRO**	**CTX**	**MEM**
*99*	*Escherichia coli*	R	S	R	S	S	S	R	R	R	S	S	S	S
*119*	*Escherichia coli*	S	R	R	S	R	S	S	R	R	S	S	S	S
*212*	*Escherichia coli*	R	S	R	S	R	S	R	R	R	S	S	S	S
*237*	*Escherichia coli	R	R	R	S	R	R	S	R	R	R	R	R	S
*253*	*Escherichia coli*	S	S	R	S	S	S	R	R	S	S	S	S	S
*275*	*Escherichia coli*	R	S	R	S	R	S	R	R	R	S	S	S	S
*300*	*Escherichia coli*	R	R	R	S	R	S	R	R	R	S	S	S	S
*28*	*K. pnuemoniae*	R	R	R	R	R	S	R	R	R	S	S	S	S
*50*	*K. pnuemoniae*	R	S	R	R	R	S	R	R	R	S	S	S	S
*101*	*K. pnuemoniae	R	S	R	R	R	S	R	R	R	R	R	R	S
*189*	*K. pnuemoniae	R	R	R	S	R	S	R	R	R	R	R	R	S
*255*	*K. pnuemoniae*	R	S	R	R	R	S	R	R	R	S	S	S	S
*215*	*K. pnuemoniae*	R	S	R	R	R	S	R	R	R	S	S	S	S
*12*	*P.aeuriginosa*	R	S	R	S	R	S	R	R	R	R	NA	NA	S
*148*	*P.aeuriginosa*	R	R	R	R	R	R	R	R	R	S	NA	NA	S
*21*	*Enterobacter* spp	S	S	S	S	R	S	S	R	R	S	**S**	S	S
*108*	*Salmonella* spp	**S**	S	S	S	S	S	S	S	R	S	S	S	S
*191*	*Citrobacter spp	R	S	R	S	R	S	S	R	R	R	R	R	S
*250*	*Citrobacter spp	R	S	R	S	R	S	S	R	R	R	R	R	S
*5*	*Proteus* spp	R	S	R	S	R	S	S	R	R	S	S	S	S
*60*	*S. aureus*	R	R	R	S	R	S	R	R	R	NA	NA	NA	S

**A**; ampicillin, **AMC**; amoxyillin/clavunate, **CIP**; ciprofloxacin, **TET**; tetracycline, **G**; gentamicin, **SXT**; sulfamethoxazole/ trimethoprim, **CRO**; ceftriaxone, **CAZ**; ceftazidime, **AK**: amikacin, **MEM**; meropenem, **NO**; Norfloxacin, **C**; chloramphenicol and **CTX**; Cefotaxime. **R**; resistant, S; sensitive. * ESBL producer, NA; Not applicable, S: Inducible resistance is expected. S; *Salmonella* spp may test susceptible in vitro but the drug is ineffective in vivo. Isolate no 60 was tested with cefoxitin and oxacillin and found to be sensitive so it is not MRSA.

### Predictors of bacteremia

An axillary temperature >38.5°C (OR =7, 95% CI = 2.2 - 14.8, p-value = 0.0001), a positive malaria blood slide (OR =5, 95% CI = 3.0 - 21.2, p-value = 0.0001) and a high neutrophils’ count (OR =21 95% CI = 5.6 - 84, p-value = 0.0001) were found to be predictors of bacteremia on both univariate and multivariate logistic regression (Table 3). A neutrophils’ count of more than 10,000 cells/liter was defined as high. Children with bacteremia had significantly higher counts of total WBC than those without bacteremia, with a median of 16.1 versus 9.1 respectively (Wilcoxon-Mann–Whitney test, p-value = 0.0001) (Table 4). Furthermore, children with bacteremia had significantly lower levels of hemoglobin than those without bacteremia, with a mean of 7.4 g/dl versus 8.4 g/dl respectively (student t-test, p-value 0.04).

**Table 3:** Showing predictors of bacteremia

**Predictive factor**	**Bacteremia**		**Univariate**		**Multivariate**	
	**YES n = 21**	**NO n = 296**	**OR (95%CI)**	**P-value**	**OR (95% CI)**	**P-value**
**Temperature**						
≤38.5°C	6 (2.6)	225 (97.4)	1			
>38.5°C	15 (17.4)	71 (82.6)	7.9 (2.9-21.2)	0.0001	13 (3.5-54)	0.0001
**Lethargic**						
No	17 (6.5)	244 (93.5)	1			
Yes	4 (7.1)	52 (92.9)	1.1 (0.4-3.4)	0.86	-	-
**Dehydration**						
No	18 (8.1)	204 (91.9)	1			
Yes	3 (3.2)	92 (96.8)	0.4 (0.1-1.3)	0.12	0.9 (0.2-3.9)	0.8
**Pneumonia**						
No	16 (6.2)	244 (93.8)	1			
Yes	5 (8.7)	52 (91.3)	1.5 (0.5-4.2)	0.48	-	-
**Convulsions**						
No	16 (5.8)	260 (94.2)	1			
Yes	5 (12.2)	36 (87.8)	2.2 (0.7-6.5)	0.13	1.3 (0.3-5.8)	0.7
**Neck stiffness**						
No	20 (6.6)	282 (93.4)	1			
Yes	1 (7.7)	12 (92.3)	1.2 (0.2-9.5)	0.88	-	-
**Antibiotics use for less than 24 hrs**						
No	7 10.0)	63 (90.0)	1			
Yes	14 (5.7)	231 (94.3)	0.6 (0.2-1.4)	0.21	-	-
**Positive Blood slide for malaria parasite**						
No	7 (3.2)	212 (96.8)	1			
Yes	13 (15.8)	69 (84.2)	5.7 (2.2-14.8)	0.0001	14 (3.5-60)	0.0001
**Sickle cell disease**						
No	15 (5.5)	259 (94.5)	1			
Yes	6 (13.9)	37 (86.1)	2.8 (1.0-7.7)	0.04	1 (0.3-4.4)	0.9
**Malnutrition**						
No	19 (7.4)	238 (92.6)	1			
Yes	2 (3.3)	58 (96.7)	0.4 (0.1-1.9)	0.26	-	-
**Neutrophils**						
≤10000 c/l	6 (2.4)	245 (97.6)	1			
>10000 c/l	15 (26.3)	42 (73.7)	14 (5.4-39.7)	0.0001	21 (5.6-84)	0.0001

**Table 4:** Showing bacteremia and complete blood count

**Complete blood count**	**Bacteremia**		**P-value**
	Positive Median (IQR)	Negative Median (IQR)	
Total WBC c/l *10^3^	16.1 (11–21.8)	9.1 (5.7-13.5)	0.0001
Lymphocytes c/l	2990 (2178–4379)	2522.5 (190–5130)	0.3935
Thrombocytes *10^9^/l	397 (226.5-529)	370 (242–507)	0.7374
*Hemoglobin g/dl	*7.4 ± 2.6	*8.4 ± 2.6	0.04

* For hemoglobin the mean ± Standard Deviation.

Of all the children enrolled into the study, 9/317 (2.8%) died; of those, 2/21 (9.5%) children had bacteremia and 7/296 (2.4%) did not have bacteremia. By multivariate logistic regression analysis, bacteremia was found to be a statistically significant predictor of in-hospital mortality. (OR = 6, 95% CI = 1.8-4.4) and P = 0.04).

## Discussion

The underfives enrolled in the present study were between 2 months and 60 months old. The age category of this study is in keeping with most of the studies done elsewhere in the world, as the children infections preventable by vaccination are prevalent before five years of age [20-22]. Of the enrolled patients, 61.5% were male, which was similar to the study done in Uganda and Malawi were male constituted 63 and 57 percent of the total children respectively [23,24]; this also matched the admission patterns at the study hospital. Only 18.9% of the under-fives presented with malnutrition. This figure was lower than the one reported in the study undertaken in Kilifi, Kenya, which quoted a prevalence of malnutrition of 25% [22]. A total of 13.6% under-fives had sickle cell disease at the time of admission which was higher than the result shown in a study done in Central Nigeria, in which only 6% of children had sickle cell disease [25]. The higher prevalence found in this context may be due to an over diagnosis of the disease, as hemoglobin electrophoresis is not routinely done to confirm sickle cell disease.

The prevalence of bacteremia among children admitted with fever was 6.6%. A study undertaken at the Muhimbili National Hospital (Tanzania) reports a prevalence of 13.9% [4]. The difference between the results could be explained by the fact that the Muhimbili study enrolled children aged 0 to 7 years and that the BACTEC MYCO/F LYTIC method for blood culture was used. This method supports the growth of fungi and fastidious bacteria including mycobacterium [4,26]. The prevalence of bacteremia in the present study was also lower than the one found by previous studies done in Mwanza and Central Nigeria which showed a prevalence of 7.4% and 20.7% respectively [19,25]. This could be explained by the fact that under-fives involved in both studies were coming directly from home, hence the use of antibiotics may have been minimal. The prevalence in our study is comparable to the study done in Kilifi, Kenya which documented a prevalence of 6% [22]. As this was a six-month cross-sectional study, seasonal variations of bacteraemia could not be addressed.

In the present study, the majority of bacteria isolated from the blood were gram-negative. Of these gram-negative bacteria, *Escherichia coli* and *Klebsiella pneumoniae* were the commonest isolates. Others gram-negatives included *Citrobacter*, *Enterobacter spp, Pseudomonas aeruginosa* and *Proteus spp*. This is contrary to others studies which reported the commonest isolate to be *Streptococcus pneumoniae, Neisseria meningitidis, Haemophilus influenza b* and Group A Streptococci [4,8]. The difference could be explained by the ongoing implementation in our setting of an extensive under-five immunization programme including the vaccination against *Haemophilus influenza* b.

The failure to isolate *Streptococcus pneumoniae* in our study could be due to self-prescription of ampicillin by patients in the community and/or to the prescription at health facilities where no routine blood culture is undertaken. It could also be due to the particular technique used in the present study. However, during the study period, *Streptococcus pneumoniae* isolates were isolated from the blood of HIV infected adults admitted in medical wards using the same blood culture techniques and the *Streptococcus pneumoniae* ATCC 49619 was used to quality control the media and incubation conditions. These facts minimize therefore the possibility of a technical problem.

The number of isolates in this cohort was too small to establish local susceptibility patterns but has provided baseline data and has stimulated the surveillance of isolates involved in blood stream infections in our setting. The gram-negative bacteria were multiply-resistant to ampicillin, co-trimoxazole, tetracycline, gentamicin, amoxicillin/clavulanate and chloramphenicol, which are locally available antibiotics. Almost all isolates were resistant to ampicillin and sulphamethaxazole/trimethoprim, which are commonly self-prescribed in the local communities [18]. Of 317 children, 247 (78%) were referred from peripheral health facilities so the resistance patterns could reflect hospital-acquired infections. However, due to a high resistance of *Escherichia coli* and *Klebsiella pneumoniae* to antibiotics commonly used by the community, as described by Msaki et al. [18], community-acquired resistance is also a possibility. The susceptibility patterns are similar to the findings reported by Muhimbili and Mwanza in Tanzania and Mozambique, which found that the majority of *Escherichia coli, Klebsiella pneumoniae* and *Salmonella spp* were resistant to gentamicin, chloramphenicol and Sulphamethaxazole/trimethoprim [4,27-29].

This study showed that a high body temperature, malaria and a high absolute neutrophils count were strong predictors of bacteremia. The study findings are similar to those previously reported [20,30,31]. The strong association between malaria and bacteremia demonstrated in this study concurred with the studies undertaken in Kenya and Malawi [22,24,32]. In contrast to previous studies, HIV was not found to predict bacteremia. This could be explained by the low prevalence of HIV infection among the group of children studied.

In the current study, the mortality rate was 2.8% and was strongly predicted by bacteremia. The presence of gram-negative bacteria might have contributed to the risk of death. As a matter of fact, a study done at the Muhimbili National Hospital inTanzania reported a mortality rate from gram-negative bloodstream infections more than the double of the one due to malaria and gram-positive bloodstream infections [4]. The mortality rate observed in the present study is lower than the one observed in other African countries such as Kenya, Zaire and Malawi [33-35]. It is also lower than the one previously reported at the Muhimbili National Hospital [4]. These results could be explained by the empirical treatment practiced at the Bugando Medical Centre (BMC), whereby children admitted were all given a second line treatment (third generation cephalosporins) though about 78% of them had already received antibiotic treatment at the peripheral health facilities before being referred to the BMC.

Among the deaths, only two patients had bacteremia due to *Pseudomonas aeruginosa* and one had malnutrition and co-infection with malaria. The two isolates of *Pseudomonas aeruginosa* were resistant to the commonly used antibiotics, i.e. first line empirical treatment, which is ampicillin and gentamicin and second line empirical treatment, which is third generation cephalosporins. The third line empirical treatment in our setting is meropenem but is often not available and, if available, is too expensive for the patients to afford. The cause of mortality was in keeping with a study done in the Gambia which found that significant risk factors for death were infection with multi-drug resistant bacteria and malnutrition [36].

Recall bias on symptoms and failure to perform antibiotics assay in body fluid and investigations of other causes of fever were the major limitations of this study. Prior antibiotic use due to self-prescription and prescription from other health care facilities may have affected the blood culture yield.

## Conclusion

A high axillary temperature, a positive malaria slide and a high absolute neutrophils’ count are independent risk factors for bacteremia among under-fives in our setting. Furthermore, most of the bacteria isolated from blood among underfives are multiply-resistant gram-negative enteric bacteria. Of all the factors under investigation, bacteremia was found to be a strong predictor of death among underfives. A large community- and hospital-based epidemiological surveillance should be conducted so that a proper and effective management of antibiotics is instituted, especially in the treatment of under-fives with a high grade fever, a positive malaria slide and a high neutrophils count. Our findings also emphasize the urgent need for surveillance data to monitor the resistance trends to commonly used antibiotic and third generation cephalosporins. Infection prevention strategies and antimicrobial stewardship to contain the further multiplication and spread of antimicrobial resistance should also be considered. It should be clearly understood that an increased use of third generation cephalosporins can result in a worsening of the situation. Third generation cephalosporins should be preserved, for as long as possible, with antimicrobial stewardship to reserve their use exclusively for patients where first-line therapy has failed. Moreover, the infection control to prevent spread of ceftriaxone -resistant organisms from one patient to another can reduce their use. The implementation of the lessons learned from third generation cephalosporins will allow the rational management of carbapenems when they will become readily available in developing countries and will prevent carbapenemases to become as rampant as ESBL’s are now.

## Competing interests

The authors declare that they have no competing interests.

## Authors’ contributions

AC participated in the design of the work, the collection of specimens, the collection of clinical data, the follow up of the patients and data analysis; SEM participated in the design and microbiological procedures, interpreted the data and prepared the first draft of the manuscript; BRK participated in data analysis and manuscript writing; AH participated in the design of the work and manuscript writing; DM participated in data interpretation and manuscript writing. All authors read and approved the final manuscript.
